# Cytokine, Chemokine, and Growth Factor Profile Characterization of Undifferentiated and Osteoinduced Human Adipose-Derived Stem Cells

**DOI:** 10.1155/2017/6202783

**Published:** 2017-05-10

**Authors:** F. Mussano, T. Genova, M. Corsalini, G. Schierano, F. Pettini, D. Di Venere, S. Carossa

**Affiliations:** ^1^CIR Dental School, Department of Surgical Sciences, UNITO, Via Nizza 230, 10126 Turin, Italy; ^2^Department of Life Sciences and Systems Biology, UNITO, Via Accademia Albertina 13, 10123 Turin, Italy; ^3^Dipartimento Interdisciplinare di Medicina, Università di Bari, Piazza Giulio Cesare 11, 70124 Bari, Italy

## Abstract

Bone is the second most manipulated tissue after blood. Adipose-derived stem cells (ASCs) may become a convenient source of MSC for bone regenerative protocols. Surprisingly, little is known about the most significant biomolecules these cells produce and release after being osteoinduced. Therefore, the present study aimed at dosing 13 candidates chosen among the most representative cytokines, chemokines, and growth factors within the conditioned media of osteodifferentiated and undifferentiated ASCs. Two acknowledged *osteoblastic* cell models, that is, MG-63 and SaOs-2 cells, were compared. Notably, IL-6, IL-8, MCP-1, and VEGF were highly produced and detectable in ASCs. In addition, while IL-6 and IL-8 seemed to be significantly induced by the osteogenic medium, no such effect was seen for MCP-1 and VEGF. Overall SaOS-2 had a poor expression profile, which may be consistent with the more differentiated phenotype of SaOs-2 compared to ASCs and MG-63. Instead, in maintaining medium, MG-63 displayed a very rich production of IL-12, MCP-1, IP-10, and VEGF, which were significantly reduced in osteogenic conditions, with the only exception of MCP-1. The high expression of MCP-1 and VEGF, even after the osteogenic commitment, may support the usage of ASCs in bone regenerative protocols by recruiting both osteoblasts and osteoclasts of the host.

## 1. Introduction

Unlike the majority of adult tissues, bone is capable to self-repair without forming scars, as most fractures demonstrate by healing spontaneously [1] or through mild surgery. Notwithstanding this inherent regenerative capacity of bone, at least one tenth of the more than 6.2 million fractures [2] occurring yearly suffer from impaired healing. In addition, inborn malformations, alveolar resorption, and critical-size bone defects resulting from severe trauma or malignant tumor resection [3] make bone the second most *transplanted* tissue after blood [4]. Treatments include grafting with both autogenous and allogenic bone, which are not without limitations [5].

Autogenous bone is widely considered the gold standard of bone grafting materials. Nevertheless, there are still some limits to the use of autogenous bone due to the donor site morbidity, the difficulty in obtaining it, and the prolonged healing time [6, 7]. Recently, autologous bone has been used for the regeneration of bony structures and defects [8]. However, autologous bone administration has been highly associated with the risk of disease transmission and immune reaction [9]. Furthermore, synthetic bone grafting materials have been produced to mimic the bone structure and to promote osteoconduction. However, fabricating and manufacturing these graft materials preclude their extensive application due to the involved primary expenses [7, 10].

One of the major goals of tissue engineering [11] is to overcome the pitfalls traditional techniques face when applied to treat large bone defects [12]. Among the three key components of each regenerative protocol, besides scaffolds and signaling molecules, cells play a paramount role. To this end, primary multipotent stem cells, along with several immortalized cell lines, have been widely used for cytocompatibility testing and osteogenic potential evaluation of biomaterials in regenerative medicine [13]. However, the heterogeneity of these cells, too often simply defined as *osteoblasts* or *osteblastic precursors*, should be carefully considered.

Albeit easy to obtain and handle, tumor-derived cell lines may present peculiar nonphysiological features [14]. For instance, osteosarcoma cell lines (SaOs-2, MG-63, and U-2 OS) differ significantly from primary osteoblasts as for immunocytochemical markers and matrix produced [15]. The most used human cell line SaOs-2 cells display a mature osteoblast phenotype and form a calcified matrix resembling woven bone [16]. SaOs-2 cells share with primary human osteoblasts a similar expression profile of cytokines, growth factors, and receptors for parathyroid hormone [17]. MG-63 cell line represents an immature osteoblast phenotype. Despite the inconsistencies about their mineralization capabilities [14], MG-63 cells have been used in long-term studies concerning cell behavior on biomaterials [18]. Notwithstanding the abovementioned pitfalls, SaOs-2 and MG-63 cells are the most studied osteoblasts.

On the other hand, primary stem cells are characterized by higher variability and are usually available in smaller amounts [19]. Although, mesenchymal stem cells deriving from bone marrow are somehow archetypic [20, 21], more recently, adipose-derived stem cells (ASCs) [22] have emerged as a viable alternative source of mesenchymal cells. As it has been exhaustively reviewed [23], ASCs are relatively abundant and easy to access and may therefore become the elective source of mesenchymal stem cells for bone regenerative protocols. Surprisingly, however, little is known about the most significant biomolecules osteo-committed cells produce and release. Therefore, the present study aimed at dosing 13 candidates chosen among the most representative cytokines, chemokines, and growth factors within the conditioned media of osteodifferentiated and undifferentiated ASCs. As a complimentary analysis, two acknowledged “osteoblastic” cell models were compared, based on their different maturation stage.

## 2. Materials and Methods

### 2.1. Cell Culture

ASCs were isolated from fat tissue obtained from three different donors as described previously [22] and maintained in Dulbecco's minimum essential medium enriched with sodium pyruvate and supplemented with 10% foetal bovine serum (FBS, Gibco Life Technologies), 100 U/ml penicillin, 100 *μ*g/ml streptomycin, and 250 ng/ml amphotericin B. The nonadherent cell population was removed after 48 h, and the adherent cell layer was washed twice with fresh medium; cells were then continuously cultured since their harvest until sixth passage. SaOs-2 (ATCC number: HTB-85) and MG-63 (ATCC number: CRL-1427) cells were, respectively, cultured in McCoy'5A (Gibco, Life Technologies) with 15% FBS (Benchmark, Gemini Bio-Products) and in Dulbecco's modified eagle's medium (DMEM, Gibco, Life Technologies) with 10% FBS. Both media were supplemented with 1% penicillin-streptomycin (MD Biomedicals, Thermo Fisher Scientific). Cells were always passaged at subconfluency to prevent contact inhibition and were kept under a humidified atmosphere of 5% CO2 in air, 37°C.

### 2.2. Detection of Interleukins, Chemokines, and Growth Factors Using Bio-Plex System

To analyze the profile of the biomolecules, cells were seeded in 96-well plates (10^3^ cells/well) in their own maintaining medium for 1 day. Afterwards, cells were incubated in RPMI in the presence of 2% FBS and 2% FBS + osteogenic factors (50 *μ*M ascorbic acid, 10 mM beta glycerophosphate, and 100 nM dexamethasone) either for 7 (T1) and 14 (T2) in the case of SaOs-2 or for 21 (T1) and 28 (T2) days in the case of MG-63 and ASCs. At the day of harvest, media were removed, cells washed twice in PBS, and fresh starving medium (RPMI 0.5% bovine serum albumin) was incubated for 2 hours. Conditioned media thus obtained were characterized, without adding any activation substances, by measuring the concentration of the following specific biomolecules: interleukin-2 (IL-2), interleukin-6 (IL-6), interleukin-8 (IL-8), interleukin-10 (IL-10), interleukin-12 (IL-12), granulocyte-colony stimulating factor (G-CSF), interferon-gamma (INF-*γ*), tumor necrosis factor-*α* (TNF-*α*), monocyte chemoattractant protein-1 (MCP-1) (CCL-2), CXCL10 chemokine (IP-10), platelet-derived growth factor (PDGF), basic-fibroblastic growth factor (bFGF), and vascular endothelial growth factor (VEGF). The flexible Bio-Plex system (Bio-Rad Laboratories, Hercules, CA, USA) was employed as previously described [24]. All samples were analyzed following the manufacturer's protocol. At least two independent repetitions in duplicate were made per sample. Concentrations of the analytes were expressed in pg/ml. A standard curve ranging on average from 0.15 pg/ml to 3700 pg/ml (High Photomultiplier Tube Setting—PMT setting) was prepared and then fitted by Bio-Plex Manager software.

### 2.3. In Vitro Osteogenic Differentiation Tests

In vitro osteogenic differentiation was performed at the same conditions described above to run a series of assays aiming at revealing established bone markers, as described elsewhere [25, 26].

#### 2.3.1. Alkaline Phosphatase (ALP) Activity Assay

Alkaline phosphatase (ALP) activity was determined using a colorimetric end point assay [27, 28], which measures the conversion of the colorless substrate p-nitrophenol phosphate (PNPP) by the enzyme ALP to the yellow product p-nitrophenol. To measure ALP activity, cells were lysed with 0.05% Triton X-100 and incubated with the reagent solution containing phosphatase substrate (Sigma-Aldrich, Milan, Italy) at 37°C for 15 min. The rate of color change corresponds to the amount of enzyme present in solution. Optical density was measured at a wavelength of 405 nm (reference 620 nm). Samples were compared against the calibration curve of p-nitrophenol standards. The final alkaline phosphatase concentration was adjusted per total protein content, to avoid biases due to the cell number. Therefore, part of the cell lysates obtained for ALP quantification was incubated with BCA™ (Thermo Fisher Scientific, Waltham, MA, USA) protein assay, following to the manufacturer's instructions. Optical density was measured at a wavelength of 570 nm, and results were adjusted to a calibration curve made by known number of cells. ALP values were determined and normalized on whole protein content at day 3 in SaOs-2 and at day 7 in MG-63 and ASCs.

#### 2.3.2. Calcium Content Assay

Cell calcium content was determined at day 14 for SaOs-2 and at day 21 for MG-63 and ASCs by Calcium colorimetric assay kit (BioVision Research Products, Mountain View, CA, USA), according to the manufacturer's protocol. The OD was measured at 575 nm within 20 minutes since preparation. A calibration curve was always made.

#### 2.3.3. Collagen and Calcium Staining

At the established time points, cells grown in six-plate wells were washed once with PBS and fixed with 4% paraformaldehyde for 10 min at room temperature. The solution was removed and cells were washed with PBS. To stain collagen, Sirius Red dye (Direct Red 80, Sigma-Aldrich) dissolved (1 mg/ml) in a saturated aqueous solution of picric acid (Sigma-Aldrich), was added to the fixed cell cultures. After kept under mild shaking for 2 hours, samples were quickly rinsed in acid water (0.5% acetic acid in pure water) and then abundantly washed with distilled water. Calcium salts were stained after von Kossa following published protocols [15]. For both picro-Sirius Red and von Kossa stains, the cultures were observed under light microscopy and representative pictures captured by an Olympus camera.

### 2.4. Statistical Analysis

Data were analysed by GraphPad Prism6 (GraphPad Software, Inc., La Jolla, CA, USA). Each experiment was repeated at least three times. Statistical analysis was performed by using the nonparametric test Wilcoxon–Mann–Whitney test. A *p* value of <0.05 was considered significant.

## 3. Results

### 3.1. Detection of Interleukins, Chemokines, and Growth Factors

**Figure 1:**
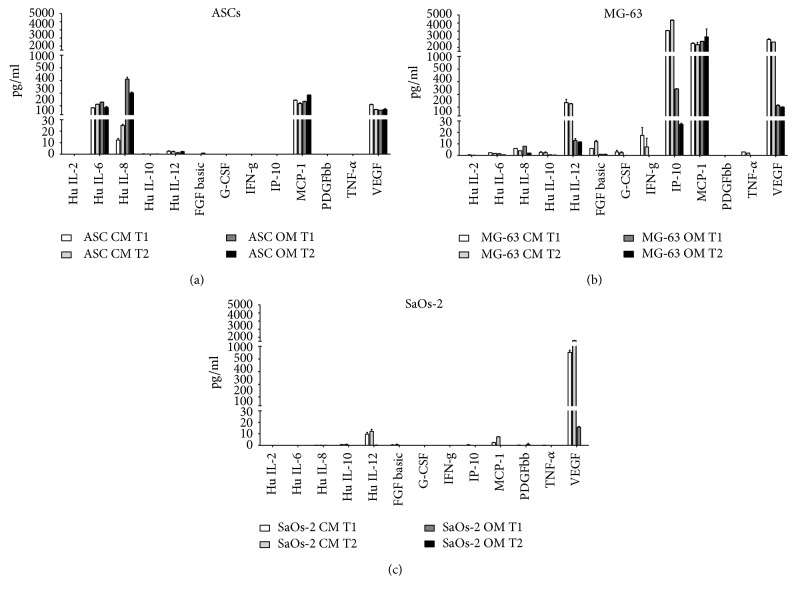
Cytokine quantification. Cytokine levels of ASCs (a), MG-63 (b), and SaOS-2 (c) measured by Bio-Plex analysis are shown. Two times (T1, T2) and two conditions (CM, OM) were considered for each cell line. For ASCs and MG-63, T1 = 21 days and T2 = 28 days; for SaOs-2, T1 = 7 days and T2 = 14 days. CM = control medium (DMEM 2% FBS); OM = osteogenic medium (see Materials and Methods).

The concentrations of interleukin-2 (IL-2), interleukin-6 (IL-6), interleukin-8 (IL-8), interleukin-10 (IL-10), interleukin-12 (IL-12), granulocyte-colony stimulating factor (G-CSF), interferon-gamma (INF-*γ*), tumor necrosis factor-*α* (TNF-*α*), monocyte chemoattractant protein-1 (MCP-1) (CCL-2), CXCL10 chemokine (IP-10), platelet-derived growth factor (PDGF), basic-fibroblastic growth factor (bFGF), and vascular endothelial growth factor (VEGF) are reported in Figure 1 for ASCs, MG-63, and SaOs-2 cells that were kept both under maintaining and differentiation media.

**Figure 2:**
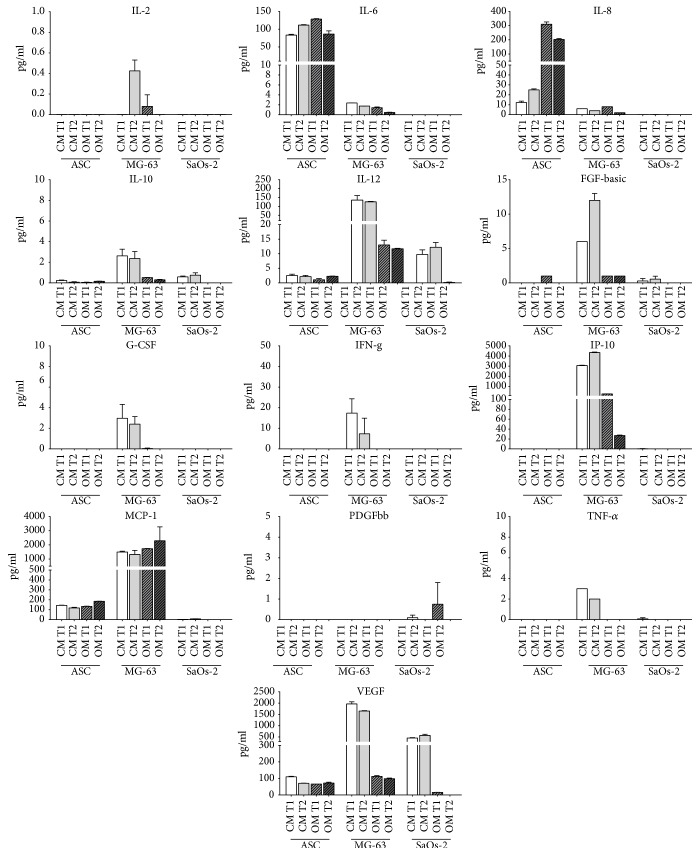
Cytokine quantification 2. Data from Bio-Plex analysis are reported as different histograms for each cytokine. In particular, the quantification of each molecule in ASCs, MG-63, and SaOs-2 is shown at T1 and T2 and in CM and OM conditions. For ASCs and MG-63, T1 = 21 days and T2 = 28 days; for SaOs-2, T1 = 7 days and T2 = 14 days. CM = control medium (DMEM 2% FBS); OM = osteogenic medium (see Materials and Methods).

Interestingly, there is a big difference in the expression pattern of interleukins, chemokines, and growth factors among different cells. ASCs produce a considerable level of IL-6, IL-8, MCP-1, and VEGF without particular variations between osteodifferentiated and control condition (with the exception of IL-8). MG-63 shows high levels of expression of IL-12, IP-10, MCP-1, and VEGF. Importantly, in osteodifferentiating conditions, the expression of IL-12, IP-10, and VEGF decreases. SaOs-2 cells show very low expression levels of the considered molecules, except for the VEGF. Notably, the osteodifferentiating medium inhibits the expression of IL-12 and VEGF in SaOs-2, similarly to MG-63 cells. To further highlight the differential expression of the considered molecules among ASCs, MG-63, and SaOs-2 cells, a panel showing the expression values for each biomolecule is reported in Figure 2.

### 3.2. In Vitro Osteogenic Differentiation Tests

**Figure 3:**
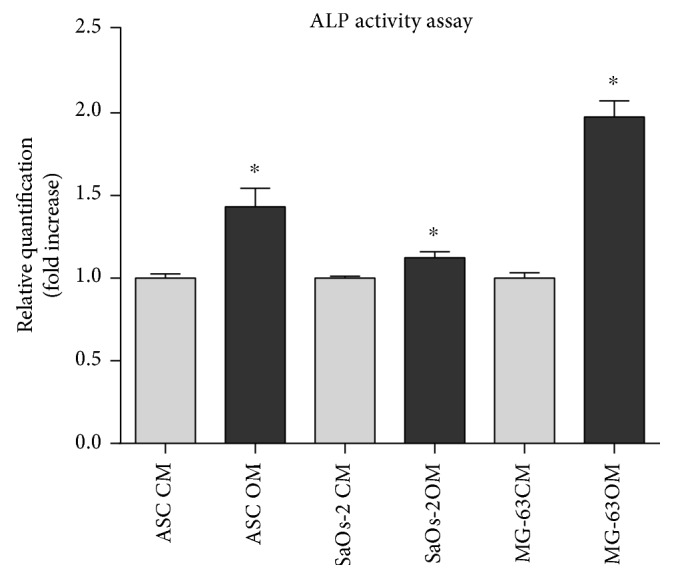
ALP activity quantification. ALP activity was evaluated recurring to a colorimetric assay. Values were normalized on whole protein content at day 3 in SaOs-2 and at day 7 in MG-63 and ASCs. For each cell type, data were normalized on control condition (CM) set as 1. OM condition significantly increase the ALP activity in each cell type. CM = control medium (DMEM 2% FBS); OM = osteogenic medium (see Materials and Methods). Statistical analysis was performed by using the Wilcoxon–Mann–Whitney test. A *p* value of <0.05 was considered significant.

**Figure 4:**
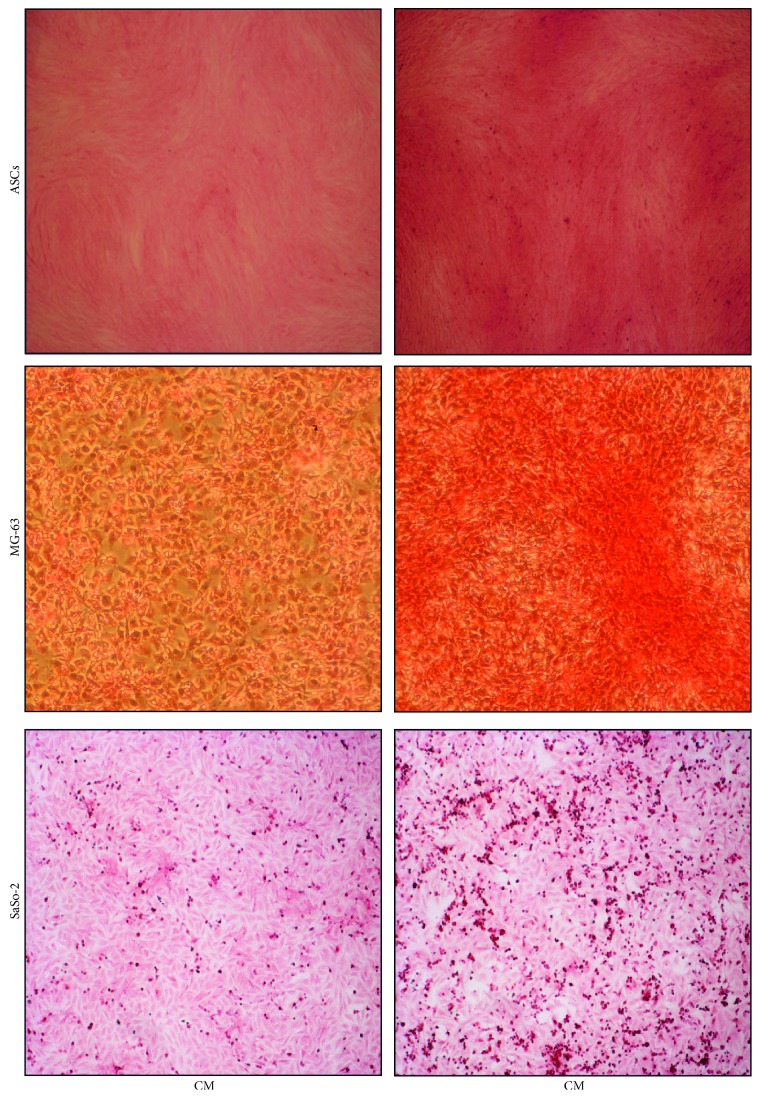
Collagen staining. Sirius Red dye staining was performed in order to show collagen deposition at day 3 in SaOs-2 and at day 7 in MG-63 and ASCs. In OM condition, the staining is more intense for each cell type. CM = control medium (DMEM 2% FBS); OM = osteogenic medium (see Materials and Methods). Images were taken at 100x magnification.

**Figure 5:**
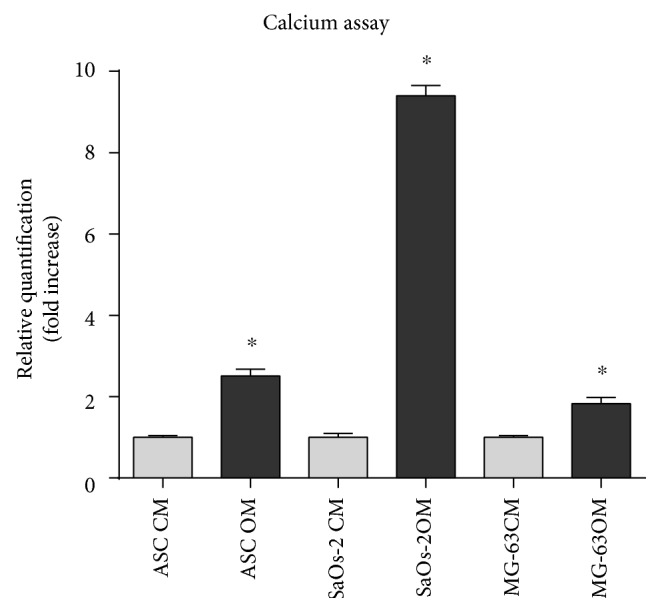
Calcium quantification. Cell calcium content was determined kit recurring to a colorimetric assay. Evaluation was performed at day 14 for SaOs-2 and at day 21 for MG-63 and ASCs. For each cell type, data were normalized on control condition (CM) set as 1. OM condition significantly increases the calcium content in each cell type with a particularly high level in SaOs-2. CM = control medium (DMEM 2% FBS); OM = osteogenic medium (see Materials and Methods). Statistical analysis was performed by using the Wilcoxon–Mann–Whitney test. A *p* value of <0.05 was considered significant.

**Figure 6:**
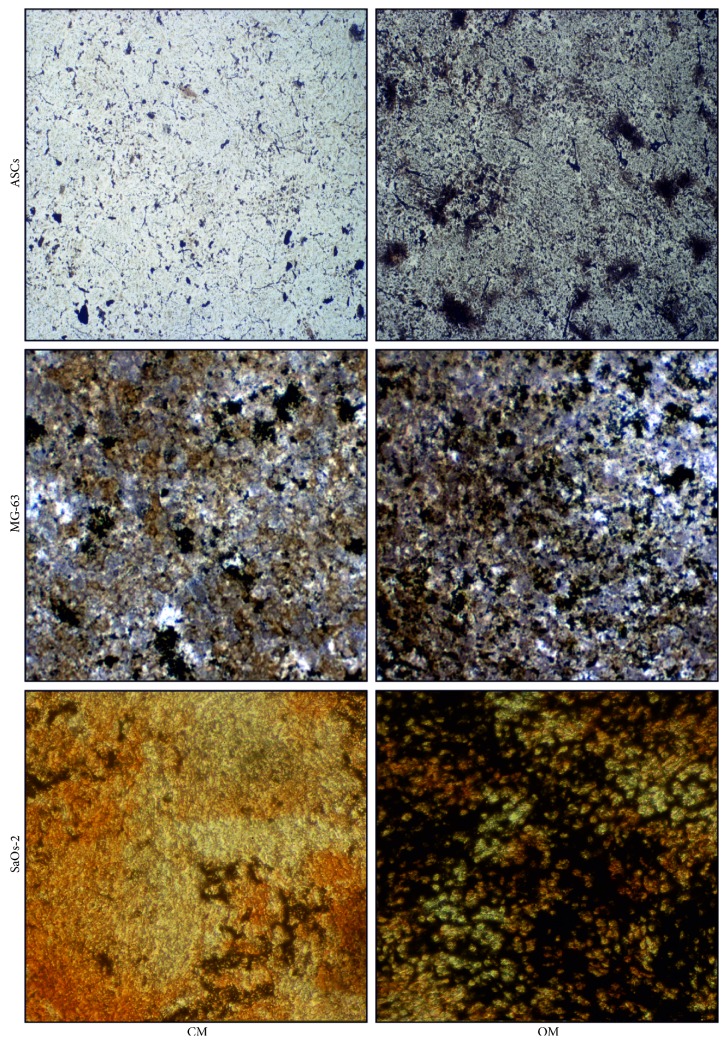
Calcium staining. Von Kossa staining was performed in order to show calcium deposition at day 14 for SaOs-2 and at day 21 for MG-63 and ASCs. In OM condition, the staining is more intense for each cell type. CM = control medium (DMEM 2% FBS); OM = osteogenic medium (see Materials and Methods). Images were taken at 100x magnification.

The osteogenic potential of the cells has been assessed at the early stage by quantifying ALP activity (Figure 3) and staining the collagen matrix through Sirius Red (Figure 4). Interestingly, the osteodifferentiating condition significantly increased the level of ALP activity for each cell type. At later stages, the extracellular calcium content was determined colorimetrically (Figure 5) and with the Von Kossa method (Figure 6). In osteodifferentiating condition, a significant increase of extracellular calcium content was found for each cell type. Collectively, the differentiating condition appeared more performing than the undifferentiated control, proving the effectiveness of the osteogenic medium.

## 4. Discussion

In the present study, the differential expression of signaling molecules among three different cell types under both osteodifferentiating and control conditions is shown for the first time. To achieve this, a highly sensitive method was used. In particular, the cellular models considered in this work are the ASC, the MG-63, and the SaOs-2 cells. Notably, the ASCs represent a particular type of mesenchymal stem cells of great potential applications in the context of bone regeneration. On the other hand, despite their ineligibility for clinical use owing to their tumor derivation [29–31], MG-63 and SaOs-2 cells were chosen to this study as they are a widely diffused and accepted in vitro model, in the field of bone biology [16, 17, 32–37]. This paper underlines also the differences in the expression variations of signaling molecules during differentiation among cells.

In 2001, Zuk et al. [22] described a putative population of multipotent stem cells isolated through the enzymatic digestion of the stromal vascular fraction of adipose tissue. Cultured over time, these adherent cells display features of multipotency; specifically, they tend to become relatively homogenous trough passages and are capable to undergo differentiation toward adipocytes, osteoblasts, and chondrocytes, under proper conditions [38]. Since this is true even when expanded from a single clone, these cells have been termed “adipose-derived stem cells” (ASCs) based on a consensus reached by the Second Annual Meeting of the International Fat Applied Technology Society [39].

Notwithstanding the huge amount of research at the in vitro and in vivo levels, the clinical usage of ASCs for bone reconstruction has been limited. It is worth mentioning the successful, although almost anecdotal, treatment of critical bone defects in humans by the seeding of ASCs into poly lactic-co-glycolic acid (PLGA) scaffolds [40] and beta-tricalcium phosphate granules [41]. Bone restoration efforts may profit from the combination with traditional techniques such as grafts and ex vivo expansion under GMP techniques [42]. Increasing interest has been focused on the biomaterials used as carriers, as described, for instance, by Mellor et al., who proposed stacked electrospun polylactic acid nanofibrous scaffolds containing tricalcium phosphate nanoparticles [43].

The actual efficacy of ASCs is, however, not solely restricted to their differentiation capacity, but it owes also a great deal to the delivery and localized secretion of signaling molecules promoting, eventually, tissue recovery. Following this research route, recent studies [43, 44] have explained the therapeutic effect of ASCs in ischemic models as a result of the release of angiogenic factors such as HGF and VEGF. Human ASCs were proven to secrete both factors constitutively [45]. Kilroy et al. [46] reported that ASCs produce angiogenic (HGF and VEGF), proinflammatory (IL-6, IL-8, IL-11, LIF, and TNF alpha), and hematopoietic-supportive cytokines (G-CSF, M-CSF, GM-CSF, and IL-7) following exposure to common inductive factors including LPS. Ribeiro and colleagues characterized the secretome of ASCs with neurologic implications [47], while Succar and coworkers profiled and compared different formulations for cell therapy of osteoarthritis [48]. Nevertheless, to the authors' surprise, the scientific literature has lacked up to now a comprehensive description of a significant range of biomolecules secreted by ASCs subjected to osteogenic differentiation, the great interest being more focused on the intracellular dynamics.

Therefore, this study focused on the detection of a representative panel of signaling molecules that ASCs, SaOs-2 cells, and MG-63 cells produce when cultured in either maintaining or osteogenic medium. Each cell type behaved differently. It is noteworthy that IL-6, IL-8, MCP-1, and VEGF were highly produced and detectable in ASCs even in the absence of any stimulus. In addition, while IL-6 and IL-8 seemed to be significantly induced by the osteogenic medium, no such effect was seen for MCP-1 and VEGF. The multiplex immunological system here adopted called Luminex® is capable to simultaneously detect and quantify up to several hundreds of analytes across multiple samples, reducing time, cost, and sample requirements in comparison to ELISA assays [49]. The capture antibodies of Luminex recognise specific analytes and are attached to microbeads with defined spectral address. The technique sensitivity thus reaches concentrations even lower than 1 pg/ml, which explains, for instance, why we report on the presence of IL-12 in ASCs contradicting Kilroy and colleagues' outcomes based on ELISA kits [46].

Overall SaOS-2 cells had a poor expression profile (only IL-12 and VEGF resulted greater than 10 pg/ml), which may be consistent with the more differentiated phenotype of SaOs-2 cells compared to ASCs and MG-63, as thoroughly reviewed elsewhere [14]. Instead, when kept in maintaining medium, MG-63 cells displayed a very rich production of IL-12, MCP-1, IP-10, and VEGF. This remarkable secretory activity was inhibited by the osteogenic conditions, except for MCP-1, a chemokine pivotal for macrophage activation and thus bone remodeling. Notably, MCP-1, which is known to be constitutively expressed in osteoblasts [50], was herein enhanced in osteodifferentiated MG-63 cells.

The high level of IP-10 quantified in MG-63 cells may be correlated to the tumor origin of the cell line [50, 51]. IP-10 was possibly produced in response to IFN-g, which was detected only in MG-63 (as shown in Figures 1 and 2). Compared to ASCs and SaOs-2, MG-63 produced also more FGF-b, although the overall level is generally low. Considering these results, it could be interesting to investigate the related TGF-b expression [52].

As noted above, contrary to MG-63 and SaOs-2 cells, VEGF did not trend downward when ASCs were osteoinduced, even though the inhibitory effect of dexamethasone, present in the osteogenic medium, is well known for endothelial and tumoral cells [53, 54]. Along with the constitutive high expression of MCP-1, the steady release of VEGF may underpin the usage of ASCs for bone regenerative protocols, where these biomolecules could contribute to recruit bone cells within the host [55–58]. Very interestingly, Hu and Olsen [55] studied bone repair in mice with a monocortical defect within the tibial cortex. Osteoblast-derived VEGF was proven to stimulate crosstalk between osteoblastic, endothelial, and hematopoietic cells in a paracrine manner, while directly affecting osteoblasts via autocrine mechanisms. The role of MCP-1 was instead investigated as for the PTH-induction during osteoclastogenesis by Li et al. [58], providing a rationale for increased osteoclast activity to initiate greater bone remodeling.

On these premises, it will be of great interest to study ASCs in a more physiologic context so as to provide more reliable and predictive results. A possible approach might consist in elucidating the behavior of ASCs in coculture systems, with endothelial cells that are known to be key players in bone formation and regeneration [5].

## 5. Conclusion

Currently, the amount of proposals for the use of ASCs in tissue repair and regeneration is impressive. The number of clinical trials evaluating the efficacy and safety of ASCs in the reconstruction and regeneration of tissues increases significantly every year. According to the clinical trials database (ClinicalTrials.gov database 2015), 122 studies are currently using ASCs [59, 60].

In particular, positive results have been obtained using autologous ASCs in clinical trials for craniofacial bone reconstruction by producing new, mature, vital, and vascularized bone [40–42, 61–63]. To date, bone regeneration is the most promising field for clinical translation of experimental ASC protocols [62]. This study supports, once more, the viability of ASCs in bone tissue engineering based on the cytokines, chemokines, and growth factors detected.
